# Clinical quantification of the integrin αvβ6 by [^18^F]FB-A20FMDV2 positron emission tomography in healthy and fibrotic human lung (PETAL Study)

**DOI:** 10.1007/s00259-019-04586-z

**Published:** 2019-12-09

**Authors:** Pauline T. Lukey, Christopher Coello, Roger Gunn, Christine Parker, Frederick J. Wilson, Azeem Saleem, Nadia Garman, Maria Costa, Stuart Kendrick, Mayca Onega, Arthur R. Kang’ombe, Allan Listanco, James Davies, Joaquim Ramada-Magalhaes, Sara Moz, William A. Fahy, Toby M. Maher, Gisli Jenkins, Jan Passchier, Richard P. Marshall

**Affiliations:** 1 GlaxoSmithKline Research and Development, Brentford, UK; 2 Present Address: Target to Treatment Consulting Ltd, Stevenage BioScience Catalyst, Stevenage, SG1 2FX UK; 3 Invicro LLC, London, UK; 4 NIHR Respiratory Clinical Research Facility, Royal Brompton Hospital, London, UK; 5 Fibrosis Research Group, National Heart and Lung Institute, Imperial College, London, UK; 6 National Institute for Health Research, Nottingham Biomedical Research Centre, Nottingham University Hospitals, Nottingham, UK

**Keywords:** Idiopathic pulmonary fibrosis, [^18^F]FB-A20FMDV2 PET/CT, αvβ6 integrin, Biomarker, Pulmonary fibrosis, PET, Clinical study

## Abstract

**Purpose:**

The RGD-integrin, αvβ6, plays a role in the pathogenesis of pulmonary fibrosis through activation of transforming growth factor beta (TGFβ). This study sought to quantify expression of αvβ6 in the lungs of healthy humans and subjects with pulmonary fibrosis using the αvβ6-selective [^18^F]FB-A20FMDV2 PET ligand.

**Methods:**

[^18^F]FB-A20FMDV2 PET/CT scans were performed in healthy subjects and those with fibrotic lung disease. Standard uptake values (SUV) and volume of distribution (*V*_T_) were used to quantify αvβ6 expression. In subjects with fibrotic lung disease, qualitative assessment of the relationship between αvβ6 expression and the distribution of fibrosis on high resolution computed tomography was conducted.

**Results:**

A total of 15 participants (6 healthy, 7 with idiopathic pulmonary fibrosis (IPF) and 2 with connective tissue disease (CTD) associated PF) were enrolled. *V*_T_ and SUV of [^18^F]FB-A20FMDV2 were increased in the lungs of subjects with pulmonary fibrosis (PF) compared with healthy subjects. Geometric mean *V*_T_ (95% CI) was 0.88 (0.60, 1.29) mL/cm^3^ for healthy subjects, and 1.40 (1.22, 1.61) mL/cm^3^ for subjects with IPF; and SUV was 0.54 (0.36, 0.81) g/mL for healthy subjects and 1.03 (0.86, 1.22) g/mL for subjects with IPF. The IPF/healthy *V*_T_ ratio (geometric mean, (95% CI of ratio)) was 1.59 (1.09, 2.32) (probability ratio > 1 = 0.988)) and the SUV ratio was 1.91 (1.27, 2.87) (probability ratio > 1 = 0.996). Increased uptake of [^18^F]FB-A20FMDV2 in PF was predominantly confined to fibrotic areas. [^18^F]FB-A20FMDV2 measurements were reproducible at an interval of 2 weeks. [^18^F]FB-A20FMDV2 was safe and well tolerated.

**Conclusions:**

Lung uptake of [^18^F]FB-A20FMDV2, a measure of expression of the integrin αvβ6, was markedly increased in subjects with PF compared with healthy subjects.

**Electronic supplementary material:**

The online version of this article (10.1007/s00259-019-04586-z) contains supplementary material, which is available to authorized users.

## Introduction

Idiopathic pulmonary fibrosis (IPF) is a chronic progressive scarring of the lungs of unknown aetiology. It results in respiratory failure and has a median untreated survival of 3 years from diagnosis [1]. Diagnosis is made by a multidisciplinary team, primarily after high-resolution computed tomography (HRCT) of the lungs. The observation of subpleural, basal honeycombing is pathognomonic for IPF and reflects the histological feature of usual interstitial pneumonia (UIP) [2]. HRCT imaging has been considered to stage IPF (Walsh 2018). Visual quantification of disease on HRCT in IPF provides prognostic information. However, this approach is hampered by its subjective nature, heterogeneity and variability. Computer-based quantification of disease on HRCT may provide more objective and reproducible data. However, this field suffers from the lack of large, well-annotated imaging datasets which hinder the development and testing of new computer-based tools, and lack of prospective trials for imaging biomarker validation. HRCT is not generally considered sensitive enough to be used as an efficacy endpoint in clinical studies, requiring years of treatment to observe a response.

There is no cure for IPF and pharmacological treatments are limited to drugs (pirfenidone and nintedanib) that slow disease progression but have significant side effects [3]. Unmet need remains high and new treatments are required. In some individuals with connective tissue disease (CTD) such as rheumatoid arthritis or systemic sclerosis, interstitial lung disease (ILD) including pulmonary fibrosis may develop with features and prognosis similar to those for IPF [4, 5].

The αvβ6 integrin is a cell surface adhesion receptor that is induced on damaged epithelium [6]. In its activated form, the αvβ6 integrin interacts with extra-cellular matrix (ECM) via ligands bearing the arginine-glycine-aspartic acid (RGD) tri-peptide sequence [7]. Binding of ECM ligands to αvβ6 promotes cell adhesion, activation of intra-cellular signalling pathways and local release of activated TGFβ from latent complexes in the matrix [7–9]. Active TGFβ mediates multiple pro-fibrotic processes resulting in progressive lung scarring, ultimately resulting in organ failure and death [10]. αvβ6 integrins have been demonstrated to play a role in the aetiology and progression of several fibrotic diseases including IPF, renal and hepatic fibrosis, as well as some cancers [11, 12]. A number of drugs targeting αvβ6 integrin across fibrotic diseases are currently in development including an inhaled small molecule inhibitor of αvβ6 under clinical development by GlaxoSmithKline R&D and an intravenous monoclonal antibody under development by Biogen (see studies registered on clinical trials.gov: NCT01371305, NCT02612051 and NCT03069989) [13]. Semiquantitative analysis of αvβ6 integrin expression in lung biopsy specimens from individuals with IPF has been shown to have potential prognostic significance, with higher levels predicting more rapid progression, and mortality [14]. Non-invasive tracking of αvβ6 integrin expression could therefore provide a mechanism to assess drug action, disease activity and prognosis.

The envelope protein of foot and mouth disease virus (FMDV) contains a 20 amino acid peptide sequence, NAVPNLRGDLQVLAQKVART (A20FMDV2) that binds the αvβ6 integrin with high affinity and selectivity [15–18]. Automated GMP-compatible radiochemistry non-clinical radiodosimetry of the A20FMDV2 peptide is described in the accompanying article [19]. [^19^F]FB-labelled A20FMDV2 has an acceptable toxicology profile, showed no adverse effects in healthy subjects and has an effective dose (0.0217 mSv/MBq) that permits multiple scans in a single subject [20]. In the murine bleomycin model, the evolution of pulmonary fibrosis is accompanied by an increased expression of αvβ6 integrins that is reproducibly quantifiable using SPECT assessment of A20FMDV2 binding [21].

In this study, we investigated the potential for [^18^F]FB-A20FMDV2 to safely and reproducibly quantify αvβ6 integrins in the lungs of healthy subjects as well as those with pulmonary fibrosis due to IPF or CTD-ILD.

## Materials and methods

### Study design

Study objectives and endpoints are presented in Table 1. Healthy participants underwent one [^18^F]FB-A20FMDV2 PET/CT scan each. Subjects with pulmonary fibrosis underwent an HRCT and two [^18^F]FB-A20FMDV2 PET/CT scans, up to 2 weeks apart, to assess test/retest variability. Healthy participant data was used to optimise scanning procedures prior to recruitment of participants with fibrotic lung disease.

**Table 1:** Objectives and endpoints

Objectives	Endpoints
Primary	
To determine the utility of [^18^F]FB-A20FMDV2 to quantify αvβ6 availability in healthy, IPF and CTD-ILD participants	Uptake and distribution of [^18^F]FB-A20FMDV2 in organs of interest (volume of distribution [V_T_], and/or standardised uptake values [SUV]).
Secondary	
To compare [^18^F]FB-A20FMDV2 uptake in areas of the lungs with varying degrees of fibrosis as determined by high resolution computed tomography (HRCT).	Qualitative assessment of the relationship between the distribution of fibrosis in the lungs from IPF and CTD-ILD participants and the uptake of [^18^F]FB-A20FMDV2.
To compare [^18^F]FB-A20FMDV2 uptake in IPF and CTD-ILD lungs versus healthy lungs.	Volume of distribution (V_T_), and/or standardised uptake values (SUV) in IPF and ILD vs healthy lungs.
To determine the reproducibility of [^18^F]FB-A20FMDV2 uptake in IPF and CTD-ILD participant lungs.	Test/re-test variability of volume of distribution (V_T_), and/or standardised uptake values (SUV).

Subjects were screened and recruited at Hammersmith Medicines Research (HMR), London, UK, and imaging assessments were conducted at Invicro, London, UK. The study was approved by the London–Brent Research Ethics Committee, UK (reference 13/LO/1792), and permission to administer radioisotopes was obtained from the Administration of Radioactive Substances Advisory Committee (ARSAC) of the UK (Ref: 630/3925/30809). The study is registered in clinical trials.gov (NCT02052297; RES116235).

### Participants

Main inclusion criteria were as follows: male subjects ≥ 45 years and female subjects ≥ 55 years at the time of signing the informed consent; a positive Allen’s test in at least one arm for arterial blood sampling; body weight ≥ 50 kg and body mass index (BMI) within the range 19.0–35.0 kg/m^2^. Additionally, self-reported healthy subjects or individuals with IPF [1] systemic sclerosis (SSc) [22] or rheumatoid arthritis (RA) [23] who had confirmed severe or progressive interstitial lung disease (ILD) on HRCT [24] were enrolled. Exclusion criteria included the following: a current history of liver disease, claustrophobia, radiation exposure in research studies greater than 10 mSv over the preceding 3 years or greater than 10 mSv in any single year, human immunodeficiency virus (HIV) or Hepatitis B/C virus positive status, anaemia (< 11 g/dL), abnormal blood coagulation profile and previous or current exposure to animals that may harbour FMDV2.

### Sample size

The sample size was estimated to provide a precision of < 50% for the parameters of interest (volume of distribution (*V*_T_) and/or standardised uptake values (SUV)). Preclinical data was available to conduct a preliminary estimate of the number of subjects that may be required to achieve this precision. Coefficients of variation (CV) for standardised uptake values (SUV) in rat tissues at various timepoints (*n* = 3 per timepoint) following administration of [^18^F]FB-A20FMV2 ranged between 3 and 150% for different tissues and incubation timepoints [19]. The mean CVs across all rodent tissues were 39–58%; for the lung, the mean CV ranged from 3 to 26%. Using these estimates for SUV variability from the rodent, the precision of the mean SUV calculated as half the width of a 95% confidence interval for the mean and expressed as a percentage of the estimated mean, for 6, 8 and 20 humans, was calculated for this study (Table 2). Thus, a sample size of 6 was likely to achieve the required precision, depending on the observed CV (%) in humans. Thus, a sample size re-estimation was planned to confirm the required sample size.

**Table 2:** Estimated precision for mean SUV

	Precision^1^ of mean SUV		
CV (%)	*N* = 6	*N* = 8	*N* = 20
39	41%	33%	18%
58	61%	48%	27%
100	105%	84%	47%

^1^Calculated as half the width of a 95% confidence interval for the mean and expressed as a percentage of the estimated mean

### Immunogenicity assay

A direct immunoassay for A20FMV2 was developed and validated [20]. Serum samples were stored at − 70 °C until assayed.

### Imaging

A20FMDV2 was labelled with fluorine-18 by conjugation of the resin bound precursor (A20FMDV2) to the prosthetic group [18F]-fluorobenzoic acid ([18F]FB), followed by acidic cleavage from the resin, purification by semi-preparative HPLC and reformulation in saline. The synthetic procedure of [18F]-FB-A20FMDV2 was adapted from a previously described method [19, 25]. An automated procedure was developed in-house using an Eckert and Ziegler Modular Lab system coupled with a semi-preparative HPLC system.

A venous cannula was inserted into a cubital or forearm vein and an arterial cannula was inserted into the radial artery under local anaesthesia. GMP grade [^18^F]FB-A20FMVD2 [19] was injected via the venous cannula and emission data acquired for up to 240 min. The arterial cannula was used to collect blood samples throughout the scan to enable the quantification of the total and unmetabolised [^18^F]FB-A20FMDV2-related radioactivity in whole blood and plasma throughout the duration of the scan. The data provided by the arterial blood samples collected was used to derive an input function for the analysis of emission PET data.

Whole blood arterial activity data was obtained from a continuous sampling system (ALLOG AB, Mariefred, Sweden), measured at a frequency of 1 Hz for the first 15 min of each scan (900 data points per scan). Manual (discrete) blood samples were withdrawn at the following timepoints: 5, 10, 15, 20, 25, 30, 40 and 50 min after scan start. Discrete samples were analysed for radioactivity in whole blood and plasma components using a Perkin Elmer (Cambridge, UK) Wizard 1470 gamma counter. A subset of the discrete plasma samples (5, 10, 15, 20, 30 and 50 min) were analysed to determine the fraction of radioactivity corresponding to the intact radiotracer compound, as opposed to radioactive metabolites.

The total effective radiation dose received was not expected to exceed 7.2 mSv for the healthy subjects who had a single PET/CT scan and 23.0 mSv for the pulmonary fibrosis participants who had two PET/CT scans as well as a high-resolution CT scan. This initial dosimetry estimate was based on a conservative approach using a dose conversion factor of 33.5 μSv/MBq injected activity based on preclinical data for the novel radioligand and a maximum administered activity of 180 MBq.

Following assessment of human dosimetry [20], the dose conversion factor was later revised downwards to 21.7 μSv/MBq, with correspondingly lower dose estimates of 5.1 mSv for the healthy and 18.8 mSv for the IPF patient group. Furthermore, following review of the quality of the images from healthy and IPF participants, the maximum administered activity for the CTD-ILD participants, was reduced from 180 to 150 MBq, thus lowering the maximum estimated radiation dose to 17.6 mSv.

### Image acquisition

Dynamic [18F]-FB-A20FMDV2 PET scans were acquired in list mode on a Siemens PET/CT system Biograph 6 TruePoint with TrueV (Siemens Healthcare, Erlangen, Germany). For the first scan, a high-resolution CT (HRCT) was achieved prior to the PET/CT. A low-dose CT scan was performed immediately before each PET study in order to estimate tissue attenuation (CT-AC). Following intravenous bolus injection of the radiotracer, dynamic PET emission data were acquired for at least 90 min (frame durations 8 × 15 s, 3 × 60 s, 5 × 120 s, 3 × 300 s, 6 × 600 s). If scans were longer than 90 min, additional 600-s frames were used. The dynamic images were reconstructed using Fourier rebinning and a 2D filtered discrete inverse Fourier transform algorithm with 5-mm isotropic Gaussian filter on a 128 × 128 matrix with 2.6 zoom giving 2-mm isotropic voxels. The dynamic images were also reconstructed using OSEM (2 iterations and 8 subsets) for visualisation purposes. Corrections were applied for attenuation, randoms and scatter.

### Image analysis

Attenuation correction CT (AC-CT) was used to semi-automatically delineate the left and right lungs. Then, a manual QC of each lung mask was performed: starting from one axial extremity, masks were displayed overlaid with the AC-CT image and non-lung labelled regions were manually removed (e.g. trachea). Morphological operations were applied to both lungs to remove 5 mm of the border to minimise any spillover of radioactivity from adjacent tissues. Finally, a correction was performed to remove potential breathing artefacts at the base of the lung and to avoid any radioactivity spillover from the liver.

Tissue fraction (TF) correction uses the low-dose attenuation correction CT scan to calculate the fraction of the lung volume that is air rather than tissue/blood [26]. As air cannot contain any radioligand, correcting for the tissue fraction yields outcome parameters that better reflect the tissue-specific uptake. The TF represents the amount of tissue remaining after the volume of air has been removed.

Time activity curves (TACs) were extracted from the dynamic [18F]FB-A20FMDV2 PET images for the left lung, right lung and total lungs (left + right lung). Static (SUV) and dynamic (*V*_T_) outcome parameters (described below) were derived from these TACs and the input function. Subsequently, SUV and *V*_T_ were corrected for tissue fraction by dividing the outcome parameter by the TF.

### Volume of distribution and standard uptake values

PET emission data and blood radioactivity data were collected for each scan and fitted to a variety of kinetic models to determine the optimal model to quantify the data. A physiological parameter of interest (*V*_T_: volume of distribution) was calculated for the tissue of interest.

*V*_T_ is a measure of the total (i.e. both non-displaceable and specific binding) distribution of the radioligand into the tissue [27] and is equivalent to an equilibrium partition coefficient. The one-tissue compartment (TC) model was selected via Akaike Information Criterion (AIC) as the most appropriate model [28]. MIAKAT^TM^ fits the one TC model to the data using non-linear optimisation techniques to estimate the individual rate constants (*K*_1_, *k*_2_), then calculates *V*_T_ from these rate constants as,

$$ {V}_T=\frac{K_1}{k_2} $$

In addition to *V*_T_, the model also estimates the volume of blood (*V*_B_) in the region of interest.

### Statistical analyses

This was an exploratory study and no formal statistical hypotheses were tested. Instead, estimation approaches were used to obtain mean values (including 95% confidence intervals) for the output parameters (i.e. *V*_T_ and SUV), following administration of [^18^F]FB-A20FMDV2 to healthy, IPF or CTD-ILD participants, and test-retest variability was assessed. SUV values reported are averaged over 10 to 30 min post injection of the tracer.

Statistical analysis of *V*_T_ and SUV using data from healthy participants and those with IPF was performed to estimate the difference in αvβ6 binding in both the combined lungs of IPF participants compared with healthy participants.

A linear mixed model with a subject random effect was fitted to the data with *V*_T_ or SUV as the response and visit (scan 1 and scan 2) and group (IPF and healthy) as the independent variables with and without tissue fraction correction. The probability ratio > 1 and probability ratio > 2 were also computed using the calculated *t* statistic and Kenward-Roger degrees of freedom [29] from the model.

When test-retest was performed, individual test-retest variability of the binding output parameters (difference scan 2–scan 1 divided by the mean of scan 1 and scan 2, expressed as a percentage) in IPF patients was calculated for each tissue of interest. Using these data, a Bland-Altman plot of difference scan 2–scan 1) against (mean of scan 1 and scan 2) was produced for each tissue of interest, and limits of agreement were computed. Estimates (95% CI) for within and between-subject variability in IPF patients were obtained from a mixed model fitted separately for each tissue of interest, including scan occasion as a fixed effect and subject as a random effect.

No statistical analysis was performed to compare data from healthy participants to subjects with CTD-ILD due to the small number of participants with this diagnosis recruited in the study.

Safety data was listed and summarised descriptively. No formal statistical analysis of safety data was conducted.

## Results

### Participants

A total of 15 participants (6 healthy, 7 with a diagnosis of IPF and 2 with a diagnosis of CTD-ILD) were enrolled and completed the study (Table 3). Of the two participants with CTD-ILD, one had RA and the other SSc. None of the subjects was withdrawn from the study. Seven participants with IPF were sufficient to achieve a precision of < 50% for the parameters of interest *V*_T_ and/or SUV and therefore recruitment was stopped. The small number of participants with CTD-ILD was due to recruitment being stopped after the study had achieved its primary endpoint in IPF subjects.

**Table 3:** Demographics of participants

Demographics	Healthy (*N* = 6)	IPF (*N* = 7)	CTD-ILD (*N* = 2)
Age in years (mean (SD))	61.2 (9.22)	70.6 (5.65)	63.5 (3.54)
Sex (*n* (%))			
Female	3 (50)	1 (14)	1 (50)
Male	3 (50)	6 (86)	1 (50)
BMI (kg/m^2^) (mean (SD))	25.35 (2.91)	24.81 (3.26)	30.70 (3.39)
Height (cm) (mean (SD))	171.5 (8.38)	175.7 (7.48)	168.0 (15.56)
Weight (kg) (mean (SD))	74.7 (10.7)	76.6 (10.6)	87.9 (25.6)

Use of prior and concomitant medications was reported in all 7 subjects with IPF and 2 subjects with CTD-fILD. Use of prior and concomitant medications was as expected in these disease populations. Four subjects with IPF were taking pirfenidone and two subjects were taking glucocorticoids (one subject with IPF for comorbid shingles and one subject for CTD).

### Clinical safety and tolerability

A total of 10 subjects out of 15 (75%) experienced AEs during the study (Table 4); none of which led to withdrawal. The most frequently reported AE was pain in extremities, which was reported in 4 subjects (27%), probably related to the arterial cannulation procedure. Each of the remaining AEs was reported in a single subject. All AEs were mild or moderate in intensity and had resolved by the follow-up visit. None of the AEs was considered by the investigator to be related to the test material (PET radioligand) administration. There were no deaths or SAEs.

**Table 4:** Summary of all adverse events by diagnosis (all subject population)

Adverse events, preferred term	Healthy (*N* = 6)	IPF (*N* = 7)	CTD-ILD (*N* = 2)	Total* (*N* = 15)
Any event, *n* (%)	5 (83)	4 (57)	1 (50)	10 (75)
Pain in extremity	3(50)	1 (14)	0	4 (27)
Back pain	0	1 (14)	0	1 (7)
Dizziness	1 (17)	0	0	1 (7)
Dysaesthesia	0	1 (14)	0	1 (7)
Headache	0	1 (14)	0	1 (7)
Restless legs syndrome	0	1 (14)	0	1 (7)
Catheter site bruise	0	1 (14)	0	1 (7)
Catheter site pain	1 (17)	0	0	1 (7)
Vessel puncture site bruise	0	0	1 (50)	1 (7)
Abdominal discomfort	1 (17)	0	0	1 (7)
Nasopharyngitis	0	1 (14)	0	1 (7)
Blood pressure increased	0	1(14)	0	1(7)

*Total is total number of subjects experiencing the event and not total number of events

### Immunogenicity

Immunogenicity was assessed by the presence of antibodies to A20FMDV2. Antibodies were observed in 3 subjects. One healthy participant had detectable antibodies prior to scanning and at follow-up. Two IPF participants had detectable antibodies prior to any scanning procedures, between the two scans and at follow-up. These data may imply previous exposure to FMDV2 and the presence of a cross-reacting antibody in subjects with IPF. All results were negative or very low (< 2 titre) on re-testing.

Since all results were observed prior to scanning as well as at follow-up, and all results remained as low titres during the study, none of these results was considered clinically significant. Antibodies were not detected in any other subject.

### PET imaging

Human whole body dosimetry and pharmacokinetics of [^18^F]FB-A20FMDV2 are reported elsewhere [20].

**Fig. 1:**
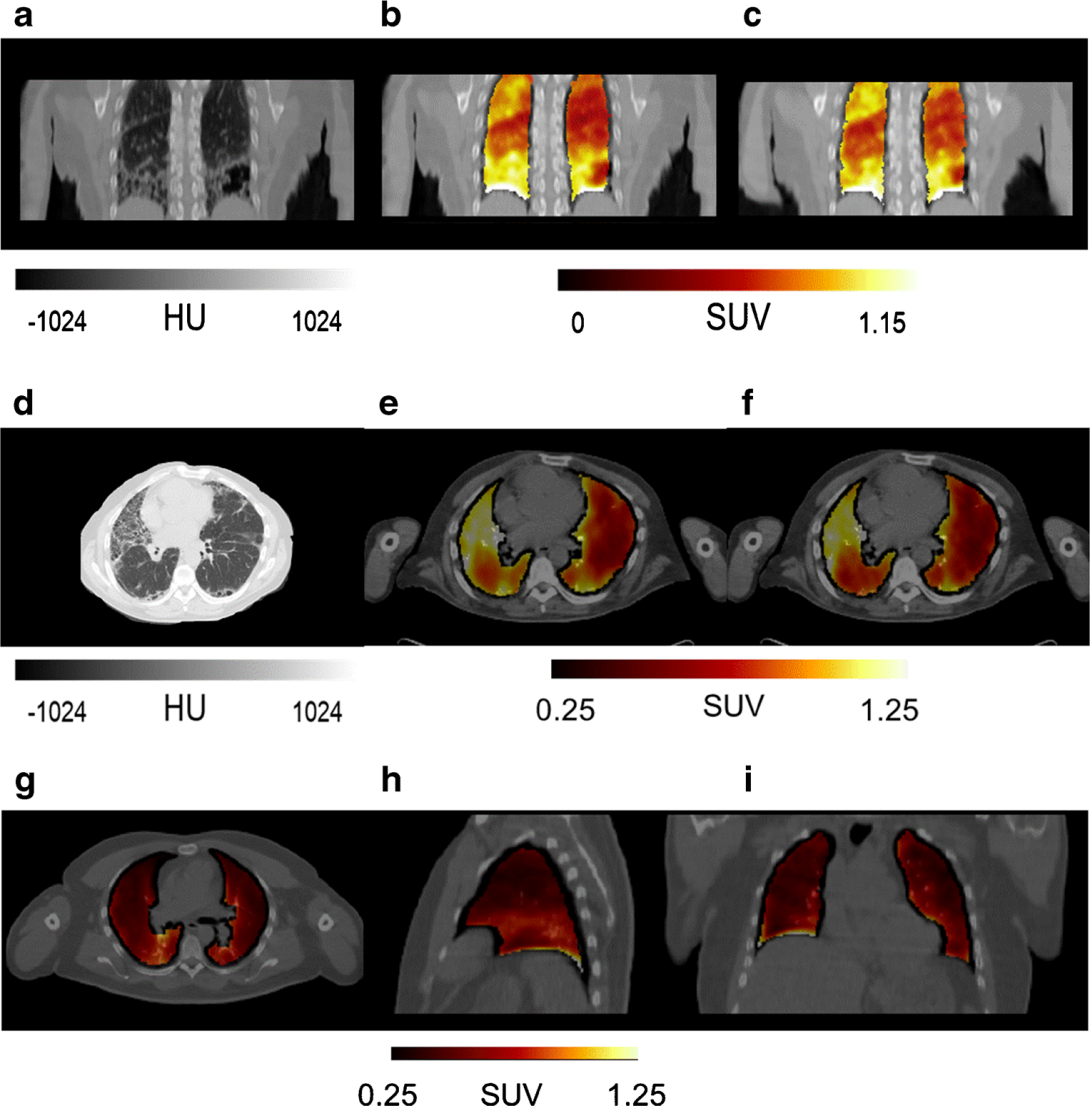
Representative images from a participant with a diagnosis of IPF and a healthy control. Coronal (A, B, C) and axial (D, E, F) images from a participant with IPF are presented. High-resolution computed tomography alone (A, D) and overlaid with the co-registered PET images for the first scan (B, E) and the second re-test scan (C, F) are presented. For comparison, axial, sagittal and coronal co-registered PET/CT images from a healthy participant are shown (G, H, J). The average SUV signal between 30 and 90 min post-dosing with [^18^F]FB-A20FMDV2 is illustrated

Dynamic [^18^F]FB-A20FMDV2 PET images were successfully acquired for six healthy participants (scan duration 56–240 min), seven IPF participants (dynamic test-retest scans of 90 min in duration) and two CTD-ILD participants (one had a single dynamic scan of 90 min in duration and the other had dynamic test-retest scans of 90 min each in duration). Injected dose (MBq), molar activity at injection time (GBq/μmol), injected mass (μg) and type of blood sampling (arterial, venous or both) are tabulated for each participant (Table 5). Due to variations in radiochemical yield, a lower mass of radioligand and/or lower radioactive dose was used for some scans. However, the administered mass of radiotracer was always sufficiently small to ensure it was at tracer concentrations, and good quality image, blood and metabolite data were acquired for all acquisitions. A qualitative assessment showed that uptake of [^18^F]FB-A20FMDV2 is low in lung tissue with a normal appearance on CT and uptake is increased in fibrotic regions as observed on CT image. Images from a representative participant with IPF are presented (Fig. 1).

**Table 5:** PET scan acquisition data including injected dose (MBq), molar activity (*A*_m_) at injection time (GBq/μmol), injected mass (μg) and type of blood sampling (arterial, venous or both)

	Injected dose (MBq)	*A*_m_ @ injection (GBq/μmol)	Injected mass (μg)	Blood samples
Healthy				
201	130.8	31.6	9.45	Arterial
202	160.9	16.9	21.7	Arterial
203	156.7	25.2	14.2	Arterial
204	146.4	20.7	16.1	Arterial
205	77.70	24.0	7.40	Arterial
206	90.50	12.0	17.2	Arterial
IPF				
301	121.0	23.2	11.9	Arterial
301	33.94	60.1	1.29	Arterial
302	142.8	27.4	11.9	Venous
302	135.7	26.4	11.8	Venous
303	130.3	26.5	11.2	Venous
303	134.9	16.2	19.1	Venous
304	78.51	78.5	2.29	Arterial
304	87.27	31.0	6.43	Arterial and venous
305	146.5	21.0	15.9	Arterial
305	94.38	18.4	11.7	Arterial and venous
306	83.41	29.9	6.38	Arterial and venous
306	125.4	116	2.47	Arterial
307	96.87	16.0	13.8	Venous
307	157.7	25.8	14.0	Venous
CTD-ILD				
401	113.9	24.5	10.7	Venous
402	124.8	20.0	14.3	Venous
402	129.0	14.1	20.9	Venous

Following intravenous administration, [18F]FB-A20FMDV2 was rapidly metabolised with 5% of parent radioactivity remaining in plasma approximately 30 min post injection.

### Time activity curves

**Fig. 2:**
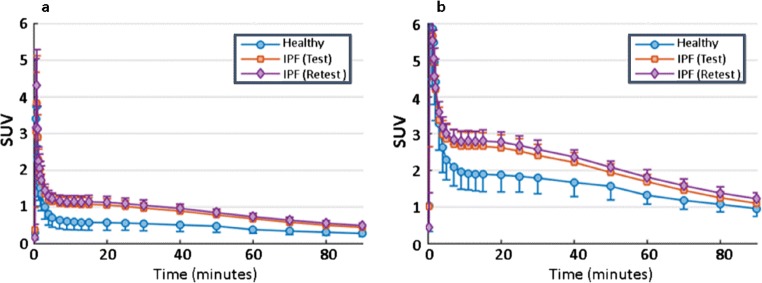
Tissue time-activity curves (TACs) in the lungs of healthy and IPF participants. **a** TACs are not corrected for tissue fraction. **b** TACs are corrected for tissue fraction. Healthy participants (HV in blue) and participants with IPF (IPF-test scan 1 red; IPF-retest scan 2 purple) are shown. Mean SUV and SD are illustrated

Dynamic PET images were used to obtain TAC for the whole lung (Fig. 2). Subjects with IPF had higher uptake than healthy subjects using both uncorrected data (Fig. 2a) and tissue fraction–corrected data (Fig. 2b).

### Volume of distribution

**Fig. 3:**
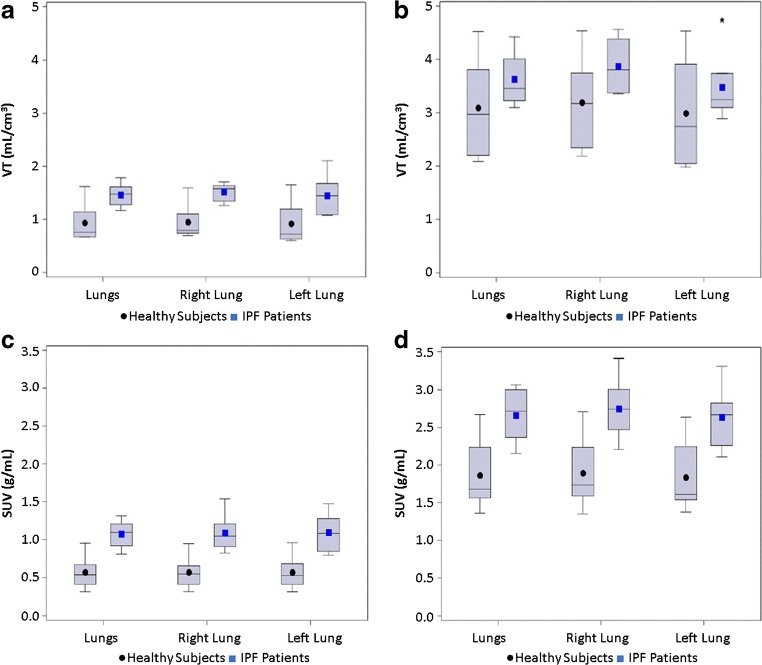
Boxplot of volume of distribution (*V*_T_) and standard uptake values (SUV) of [^18^F]FB-A20FMDV2 in lungs. **a** Uncorrected *V*_T_ data, **b***V*_T_ corrected for tissue fraction, **c** uncorrected SUV data, and **d** SUV corrected for tissue fraction. Note that values for participants with IPF are a mean of scan 1 and scan 2

Geometric mean values (95% CI of geomean) for volume of distribution (*V*_T_) without tissue fraction correction were 0.88 (0.60, 1.29) mL/cm^3^ for healthy participants, and 1.40 (1.22, 1.61) mL/cm^3^ for subjects with IPF (Fig. 3a). Geometric mean values for *V*_T_ with tissue fraction correction were 2.98 (2.17, 4.09) mL/cm^3^ for healthy participants and 3.49 (3.11, 3.91) mL/cm^3^ for participants with IPF (Fig. 3b). *V*_T_ was higher in subjects with IPF compared with healthy subjects, regardless of tissue fraction correction (Fig. 3a and b).

### Standardised uptake value

Geometric mean values for SUV without tissue fraction correction were 0.54 (0.36, 0.81) g/mL for healthy subjects and 1.03 (0.86, 1.22) g/mL for subjects with IPF (Fig. 3c). Geometric mean values for SUV with tissue fraction correction were 1.82 (1.40, 2.36) g/mL for healthy subjects and 2.56 (2.22, 2.97) g/mL for subjects with IPF (Fig. 3d).

### Analysis of left and right lung separately

*V*_T_ and SUV were higher in participants with IPF when measured in the whole lung as well as in the individual left and right lungs (Fig. 3).

### *V*_T_ and SUV changes in participants with IPF vs. healthy participants

**Fig. 4:**
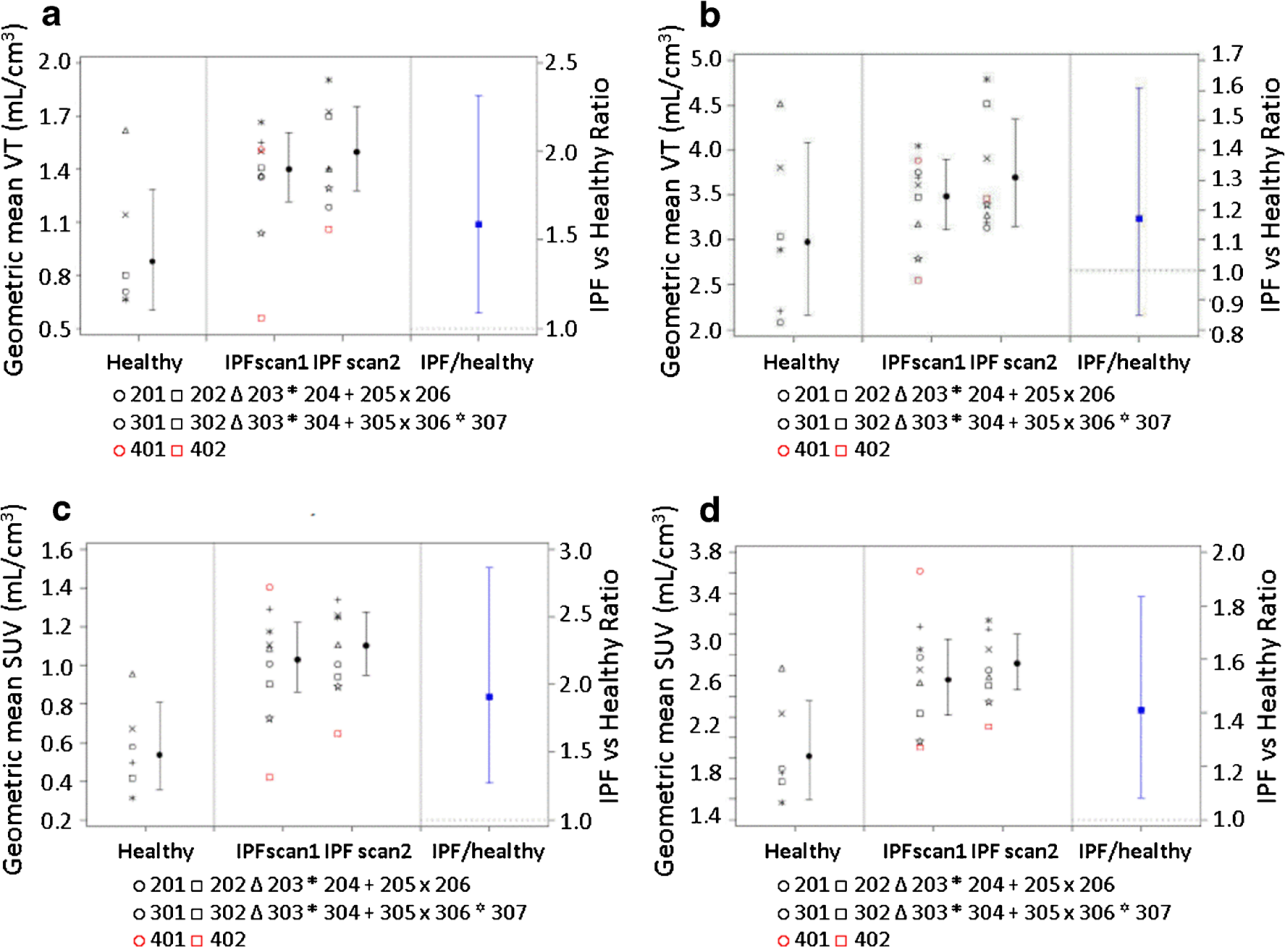
Geometric means and adjusted IPF/healthy ratio of *V*_T_ and SUV of [^18^F]FB-A20FMDV2 in lungs of healthy and IPF participants. Data from individual participants is presented (scan 1 from healthy participants numbers 201–206, scans 1 and 2 from IPF participants numbers 301–307) in black and CTD-ILD participants numbers 401 and 402 in red). **a**, **b** Geometric mean *V*_T_ and 95% CI are illustrated using the left-hand axes. **c**, **d** Geometric mean SUV and 95% CI are illustrated using the left-hand axes. Adjusted IPF/healthy ratio and 95% CI are shown on the right-hand axes in blue. **a**, **c** Not corrected for tissue fraction. **b**, **d** Corrected for tissue fraction. Note that the data from CTD-ILD participants did not contribute to the calculation of the geometric means or the adjusted IPF/Healthy ratio

Geometric means and adjusted IPF/healthy ratio of *V*_T_ and SUV of [^18^F]FB-A20FMDV2 in lungs of healthy and IPF participants without (Fig. 4a and c respectively) and with (Fig. 4b and d respectively) tissue fraction correction are illustrated.

Without tissue fraction correction, the adjusted geometric mean *V*_T_ of the IPF/healthy ratio (95% CI of ratio) estimated from the model was reported as 1.59 (1.09, 2.32) and was considered statistically significant at the 5% level. The probability ratio > 1 = 0.988 and probability ratio > 2 = 0.096 was also computed. Similarly, with tissue fraction correction, the adjusted geometric mean *V*_T_ of the IPF/healthy ratio (95% CI of ratio) was reported as 1.17 (0.85, 1.61) and was not considered statistically significant at the 5% level. The probability ratio > 1 = 0.860 and probability ratio > 2 = 0.003 were also computed).

Without tissue fraction correction, the adjusted geometric mean SUV of the IPF/healthy ratio (95% CI of ratio) was reported as 1.91 (1.27, 2.87) and was considered statistically significant at the 5% level (probability ratio > 1 = 0.996, probability ratio > 2 = 0.398). With tissue fraction correction, the adjusted geometric mean SUV of the IPF/healthy ratio (95% CI of ratio) was reported as 1.41 (1.08, 1.84) and was considered statistically significant at the 5% level (probability ratio > 1 = 0.991 and probability ratio > 2 = 0.008).

One of the healthy participants had *V*_T_ and SUV uptake values that were higher than the other healthy subjects and within the observed range for IPF participants. Whilst we did not acquire diagnostic quality HRCT images for the healthy participants, further examination of this participant’s low dose AC-CT showed diffuse reticular shadowing consistent with interstitial lung disease. The participant was referred for appropriate clinical assessment and follow-up.

### *V*_T_ and SUV test/re-test variability within participants with IPF

**Fig. 5:**
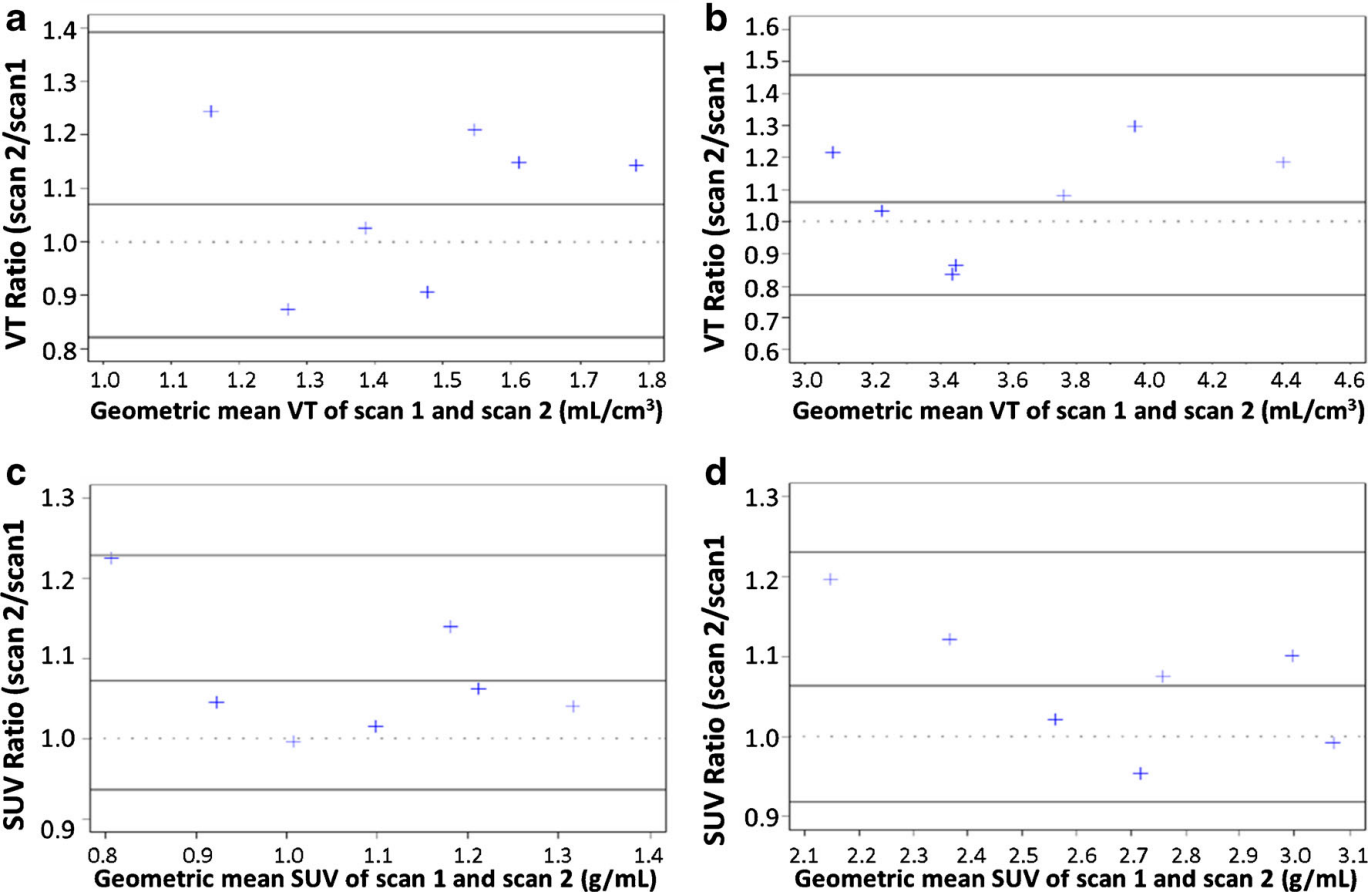
Bland-Altman plots of *V*_T_ and SUV to assess reproducibility of [^18^F]FB-A20FMDV2 combined lung scans in IPF participants. **a***V*_T_ without tissue fraction correction. **b***V*_T_ with tissue fraction correction. **c** SUV without tissue fraction correction and **d** with tissue fraction correction

Figure 5 presents Bland-Altman plots of *V*_T_ and SUV to assess reproducibility of scans in both lungs combined for [^18^F]FB-A20FMDV2 in participants with IPF. The mean difference of the *V*_T_ and SUV (scan 2–scan 1) was in the range of 0 to 0.3 and 0 to 0.1 respectively, with and without tissue fraction correction. The geometric mean ratio of the *V*_T_ and SUV (scan 2/scan 1) with and without tissue fraction correction were not statistically significantly different from 1 at the 5% level.

### Uptake in lungs of CTD-ILD

Only 2 participants with a diagnosis of CTD-ILD were recruited. The participant with RA-ILD had only 1 scan, and the participant with SSc-ILD had 2 scans. *V*_T_ and SUV for SSC-ILD were at the lower end of the range for both scans (SUV scan 1 = 0.42 g/mL and SUV scan 2 = 0.65 g/mL; *V*_T_ scan 1 = 0.56 and *V*_T_ scan 2 = 1.06). It may be significant that the HRCT of this participant with SSc-ILD had mild fibrotic changes in the lung bases posteriorly and very mild changes anteriorly. For RA-ILD, *V*_T_ and SUV were at the higher end of the range for scan 1 (SUV scan 1 = 1.40 mL/cm^3^ and *V*_T_ scan 1 = 1.51 mL/cm^3^). This participant with RA-ILD had severe fibrotic changes in all lobes on HRCT.

## Discussion

[^18^F]FB-A20FMDV2 is a potent, selective ligand for integrin αvβ6, as shown by in vitro cell-free and cellular binding assays [18, 30, 31] and by in vivo animal models of cancer [25, 32] and pulmonary fibrosis [33, 21]. In addition, it has been tested in a first dose in human study of the safety, tolerability, biodistribution, radio-dosimetry and immunogenicity of [^18^F]FB-A20FMDV2 [20]. Visual inspection of these PET images from healthy humans suggested that uptake of radioactivity was observed in the thyroid, salivary glands, liver, stomach wall, spleen, ureters and bladder [20]. Immunohistochemistry and SPECT imaging of healthy rodent tissues revealed that αvβ6 is expressed constitutively in the epithelium of the gall bladder, stomach wall, duodenum, ileum, colon, and at lower levels in the lungs and skin [34, 35]; largely supporting the observed expression in healthy humans [20].

In the current clinical study, [^18^F]FB-A20FMDV2 PET/CT was used to selectively image and quantify αvβ6 integrins in the lungs of healthy individuals and those with established fibrotic lung disease. As anticipated based on published immunohistochemistry of lung biopsies from subjects with IPF [14], we demonstrate increased levels of αvβ6 integrin expression in the lungs of subjects with IPF compared with lungs of healthy subjects. This increased expression of αvβ6 integrin was generally observed in fibrotic regions of the lung. The [^18^F]FB-A20FMDV2 signal was shown to be reproducible over a 2-week period, indicating its potential as a measure of target engagement in early phase drug development studies.

Although αvβ6 integrins are induced on epithelial cells in response to damage, detectable background levels of expression were observed in healthy human lungs, as previously described in mice [21]. In one healthy, clinically asymptomatic participant however, an unexpectedly high PET signal was observed that, together with the observation of possible reticular shadowing on the AC-CT, suggested further investigation and assessment of possible lung pathology. The identification of asymptomatic interstitial lung abnormalities (ILAs) in screened healthy populations is an emerging problem in clinical trials and a proportion of these cases may progress towards IPF [36]. It is possible that identification of pathogenic pathways such as increased levels of the αvβ6 integrin may help determine the natural history of this condition and although no overt disease was observed in this volunteer, the identification of radiological and molecular abnormalities would suggest continued careful follow-up is indicated. No further information on this participant is currently available.

Comparison of lung-based PET signals between healthy and pulmonary fibrosis subjects is complicated by regional differences in air and tissue fraction due to the fibrosis. Tissue fraction (TF) correction is used to define the volume of air (which cannot contain any radioligand) in the lung using CT scan and removes this contribution from the analysis. The lungs of IPF subjects have a smaller air fraction than healthy subjects especially in the fibrotic regions; hence, an increased PET signal could be observed due to the density change even in the absence of altered tissue uptake [37]. In this study, even after TF correction, a smaller, more variable, but still significant increase in the SUV was observed in pulmonary fibrosis. The kinetic modelling required to derive *V*_T_ also increases the variability, and these two sources of increased variability together are likely to explain why the difference in tissue-fraction-corrected *V*_T_ between fibrotic and healthy lungs did not meet the criterion for significance. On the other hand, the test-retest reproducibility would suggest that TF correction is not required in short-term studies using within-subject comparisons, because tissue density is unlikely to change over a few weeks.

Individuals with usual interstitial pneumonia (UIP) pattern on HRCT and an underlying diagnosis of connective tissue disease do not have IPF. However, due to the similar HRCT pattern and underlying histopathology of UIP, there may be similarities in the underlying mechanisms of fibrosis. We show here that αvβ6 integrin expression can be measured by PET scan in these subjects and is generally localised to the fibrotic regions. As only two subjects with CTD-ILD were included in the study, it is not possible to robustly compare PET signals between CTD-ILD and IPF. Of the two CTD-ILD subjects, the one with SSc-ILD had relatively low levels of αvβ6 expression whilst the subject with RA-ILD had relatively high levels. These differences may be due to sampling bias in such a small sample, may be related to the observed extent of fibrotic disease in the lungs on HRCT, or to differing mechanisms of fibrosis between SSc and RA.

Other PET ligands are under development to quantify expression levels of the integrin αvβ6. Cystine knot peptides have been conjugated with ^18^F-fluorobenzoate, injected into mice xenografted with αvβ6-positive or αvβ6-negative pancreatic tumours and PET scans conducted [38]. Target specificity was confirmed by low tumour uptake in αvβ6-negative tumours compared with high uptake in αvβ6-positive tumours. These cystine knot peptide tracers show translational promise for molecular imaging of integrin αvβ6 overexpression in pancreatic and other cancers [38]. In fact, [^18^F]FP-R01-MG-F2, an αvβ6-specific cysteine knot peptide is under development in a clinical study in 10 subjects with IPF (NCT03183570). It is currently premature to determine the relative benefit of each of the αvβ6 integrin PET ligands that are under development, but emerging data may provide insight into the optimal use of these tools.

There are limitations to the current study, such as the small sample size, the observed increased PET signal in a ‘healthy’ subject and the complication of tissue density. However, the data provides sufficient encouragement for ongoing testing of the utility of this ligand in a variety of clinical settings. A first time in human (FTIH) study of GSK3008348 (an inhibitor of integrin α_v_β_6_) in healthy volunteers and idiopathic pulmonary fibrosis patients has completed and the manuscript is in preparation. This study used [^18^F]FB-A20FMDV2 to explore target engagement in the lungs of IPF subjects (NCT02612051). Another clinical study using [18F]FB A20FMDV2 to evaluate αvβ6 integrin expression in the lungs of patients receiving pulmonary radiotherapy to treat non-small cell lung cancer (NSCLC) has been completed [39, 40]. In addition, a study in liver disease is also underway and will be reported separately.

In conclusion, we have developed a non-invasive imaging technique using [^18^F]FB-A20FMDV2 to quantify expression of αvβ6 integrin in the lungs of subjects with fibrotic interstitial lung disease. It is likely that PET approaches such as this will have far-reaching implications in the development of novel integrin targeting therapies for fibrotic diseases.

## Electronic supplementary material

ESM 1 (DOCX 18 kb)

## References

[1] Raghu G, Collard HR, Egan JJ, Martinez FJ, Behr J, Brown KK (2011). An official ATS/ERS/JRS/ALAT statement: idiopathic pulmonary fibrosis: evidence-based guidelines for diagnosis and management. Am J Respir Crit Care Med.

[2] Raghu G. Idiopathic pulmonary fibrosis: guidelines for diagnosis and clinical management have advanced from consensus-based in 2000 to evidence-based in 2011. Eur Respiratory Soc. 2011. 10.1183/09031936.00017711 21454891

[3] Ogura T, Taniguchi H, Azuma A, Inoue Y, Kondoh Y, Hasegawa Y et al. Safety and pharmacokinetics of nintedanib and pirfenidone in idiopathic pulmonary fibrosis. Eur Respir J. 2014. 10.1183/09031936.00198013 25504994

[4] Nicholson AG, Colby TV, DuBois RM, Hansell DM, Wells AU (2000). The prognostic significance of the histologic pattern of interstitial pneumonia in patients presenting with the clinical entity of cryptogenic fibrosing alveolitis. Am J Respir Crit Care Med.

[5] Walsh SL, Sverzellati N, Devaraj A, Keir GJ, Wells AU, Hansell DM. Connective tissue disease related fibrotic lung disease: high resolution computed tomographic and pulmonary function indices as prognostic determinants. Thorax. 2013:thoraxjnl-2013-203843. 10.1136/thoraxjnl-2013-203843 24127020

[6] Williamson JD, Sadofsky LR, Hart SP (2015). The pathogenesis of bleomycin-induced lung injury in animals and its applicability to human idiopathic pulmonary fibrosis. Exp Lung Res.

[7] Munger JS, Huang X, Kawakatsu H, Griffiths MJD, Dalton SL, Wu J (1999). The integrin (alpha)v(beta)6 binds and activates latent TGF(beta)1: a mechanism for regulating pulmonary inflammation and fibrosis. Cell.

[8] Annes JP, Rifkin DB, Munger JS (2002). The integrin (alpha)V(beta)6 binds and activates latent TGF(beta)3. FEBS Lett.

[9] Henderson NC, Sheppard D (2013). Integrin-mediated regulation of TGF + ¦ in fibrosis. Biochim Biophys Acta Mol basis Dis.

[10] Thomas BJ, Kan OK, Loveland KL, Elias JA, Bardin PG (2016). In the shadow of fibrosis: innate immune suppression mediated by transforming growth factor-β. Am J Respir Cell Mol Biol.

[11] Maubant S, Cruet-Hennequart S, Dutoit S, Denoux Y, Crouet H, Henry-Amar M (2005). Expression of α V-associated integrin β subunits in epithelial ovarian cancer andits relation to prognosis in patients treated with platinum-based regimens. J Mol Histol.

[12] Elayadi AN, Samli KN, Prudkin L, Liu Y-H, Bian A, Xie X-J (2007). A peptide selected by biopanning identifies the integrin αvβ6 as a prognostic biomarker for nonsmall cell lung cancer. Cancer Res.

[13] Chakraborty S, Chopra P, Ambi SV, Dastidar SG, Ray A (2014). Emerging therapeutic interventions for idiopathic pulmonary fibrosis. Expert Opin Investig Drugs.

[14] Saini G, Porte J, Weinreb PH, Violette SM, Wallace WA, McKeever TM (2015). avb6 integrin may be a potential prognostic biomarker in interstitial lung disease. Eur Respir J.

[15] Jackson T, Sheppard D, Denyer M, Blakemore W, King AMQ (2000). The epithelial integrin αvβ6 is a receptor for foot-and-mouth disease virus. J Virol.

[16] Monaghan P, Gold S, Simpson J, Zhang Z, Weinreb PH, Violette SM (2005). The αvβ6 integrin receptor for foot-and-mouth disease virus is expressed constitutively on the epithelial cells targeted in cattle. J Gen Virol.

[17] Burman A, Clark S, Abrescia NGA, Fry EE, Stuart DI, Jackson T (2006). Specificity of the VP1 GH loop of foot-and-mouth disease virus for αv integrins. J Virol.

[18] Slack RJ, Hafeji M, Rogers R, Ludbrook SB, Marshall JF, Flint DJ (2016). Pharmacological characterization of the αvβ6 integrin binding and internalization kinetics of the foot-and-mouth disease virus derived peptide A20FMDV2. Pharmacology..

[19] Onega M, Parker CA, Coello C, Rizzo G, Keat N, Ramada-Magalhaes J et al. Preclinical evaluation of [18F]IMAFIB as a selective marker for αVβ6 integrin using positron emission tomography in vivo. TBD. 2018;in progress. 10.1007/s00259-019-04653-5 PMC7075836 31897589

[20] Keat N, Kenny J, Chen K, Onega M, Garman N, Slack RJ et al. A first time in human, microdose, positron emission tomography study of the safety, immunogenicity, biodistribution and radiation dosimetry of [18F] FB-A20FMDV2 for imaging the integrin αvβ6. J Nucl Med Technol. 2018:jnmt. 117.203547. 10.2967/jnmt.117.203547 29438002

[21] John AE, Luckett JC, Tatler AL, Awais RO, Desai A, Habgood A (2013). Preclinical SPECT/CT imaging of alphavbeta6 integrins for molecular stratification of idiopathic pulmonary fibrosis. J Nucl Med.

[22] van den Hoogen F, Khanna D, Fransen J, Johnson SR, Baron M, Tyndall A (2013). Classification criteria for systemic sclerosis: an ACR-EULAR collaborative initiative. Arthritis Rheum.

[23] Aletaha D, Neogi T, Silman AJ, Funovits J, Felson DT, Bingham CO (2010). 2010 rheumatoid arthritis classification criteria: an American College of Rheumatology/European League Against Rheumatism collaborative initiative. Arthritis Rheum.

[24] Vij R, Strek ME (2013). Diagnosis and treatment of connective tissue disease-associated interstitial lung disease. CHEST J.

[25] Hausner SH, DiCara D, Marik J, Marshall JF, Sutcliffe JL (2007). Use of a peptide derived from foot-and-mouth disease virus for the noninvasive imaging of human cancer: generation and evaluation of 4-[18F] fluorobenzoyl A20FMDV2 for in vivo imaging of integrin αvβ6 expression with positron emission tomography. Cancer Res.

[26] Lambrou T, Groves AM, Erlandsson K, Screaton N, Endozo R, Win T (2011). The importance of correction for tissue fraction effects in lung PET: preliminary findings. Eur J Nucl Med Mol Imaging.

[27] Innis R, Vincent JC, Jacques D, Masahiro F, Albert G, Roger NG (2007). Consensus nomenclature for in vivo imaging of reversibly binding radioligands. J Cereb Blood Flow Metab.

[28] Akaike H, Parzen E, Tanabe K, Kitagawa G (1998). A New Look at the Statistical Model Identification. Selected papers of Hirotugu Akaike.

[29] Kenward Michael G., Roger James H. (1997). Small Sample Inference for Fixed Effects from Restricted Maximum Likelihood. Biometrics.

[30] Rowedder JE, Ludbrook SB, Slack RJ (2017). Determining the true selectivity profile of αv integrin ligands using radioligand binding: applying an old solution to a new problem. SLAS Discov.

[31] Gower E, Wilkinson A, Morrison V, Nanthakumar C, Slack R (2016). P109 high affinity engagement of the αvβ6 integrin induces degradation: a novel mechanism for sustained inhibition of pro-fibrotic TGFβ activation. Q J Med.

[32] Hausner Sven H., Kukis David L., Gagnon M. Karen J., Stanecki Catherine E., Ferdani Riccardo, Marshall John F., Anderson Carolyn J., Sutcliffe Julie L. (2009). Evaluation of [ 64 Cu]Cu-DOTA and [ 64 Cu]Cu-CB-TE2A Chelates for Targeted Positron Emission Tomography with an α v β 6 -Specific Peptide. Molecular Imaging.

[33] John A, Luckett J, Awas R, Habgood A, Ludbrook S, Blanchard A (2012). Targeted in vivo imaging of the αvβ6 integrin in mice with bleomycin-induced lung fibrosis. Thorax.

[34] Saha A, Ellison D, Thomas GJ, Vallath S, Mather SJ, Hart IR (2010). High-resolution in vivo imaging of breast cancer by targeting the pro-invasive integrin alphavbeta6. J Pathol.

[35] Saha A. The development and characterisation of peptides to image αvβ6 in cancer: Queen Mary University of London; 2011.

[36] Hunninghake GM. Interstitial lung abnormalities: erecting fences in the path towards advanced pulmonary fibrosis. Thorax. 2019:thoraxjnl-2018-212446. 10.1136/thoraxjnl-2018-212446 PMC6475107 30723182

[37] Chen DL, Cheriyan J, Chilvers ER, Choudhury G, Coello C, Connell M (2017). Quantification of lung PET Images: challenges and opportunities. J Nucl Med.

[38] Hackel BJ, Kimura RH, Miao Z, Liu H, Sathirachinda A, Cheng Z (2013). 18F-Fluorobenzoate–labeled cystine knot peptides for PET imaging of integrin αvβ6. J Nucl Med.

[39] Azeem S, Helo Y, Searle G, Win Z, Cook J, Gunn R (2019). Integrin-PET uptake evaluationj in patients receiving pulmonary radiotherapy.

[40] Saleem A, Helo Y, Searle G, Dekaj F, Cook J, Win Z (2019). Imaging radiotherapy-induced pulmonary fibrogenic changes with integrin-PET.

