# Supplementary material for: Clinical quantification of the integrin αvβ6 by [18F]FB-A20FMDV2 positron emission tomography in healthy and fibrotic human lung (PETAL Study)
Source: Eur J Nucl Med Mol Imaging. 2019 Dec 9;47(4):967–79. doi: 10.1007/s00259-019-04586-z (PMC7075837; doi:10.1007/s00259-019-04586-z)
Supplement: Supplementary file 1 — (DOCX 18 kb) [file 259_2019_4586_MOESM1_ESM.docx]

# Appendix 1: Supplementary material

Qualitative comparison between CT and PET images of subjects 301-307 had IPF, subject 401 had fILD associated with Rheumatoid arthritis and subject 402 had fILD associated with systemic sclerosis.

| **Study ID** | **000301 Visit 1 scan 01 Apr 2014** |
| --- | --- |
| CT finding | Fibrotic changes R>L, predominantly both lung bases, RLL and RML |
| PET finding | Tracer uptake in areas of fibrosis, but not bullae |
| Any areas of fibrotic change on CT where increase radiotracer uptake not seen | No |
| Any areas of radiotracer uptake on PET, where CT fibrotic changes not seen | No, but there is PET uptake in RUL posteriorly, where there is only subtle fibrotic changes |

| **Study ID** | **000301. Visit 2 scan 08 Apr 2014** |
| --- | --- |
| CT finding | No change from Visit 1 |
| PET finding | No change from Visit 1 |
| Any areas of fibrotic change on CT where increase radiotracer uptake not seen | No change from Visit 1 |
| Any areas of radiotracer uptake on PET, where CT fibrotic changes not seen | No change from Visit 1 |

| **Study ID** | **000302. Visit 1 scan 22 Apr 2014** |
| --- | --- |
| CT finding | Mild peripheral fibrotic changes in all lobes. |
| PET finding | See below |
| Any areas of fibrotic change on CT where increase radiotracer uptake not seen | Yes – lingular LUL segment, RLL and LLL posteriorly. RUL antero-laterally |
| Any areas of radiotracer uptake on PET, where CT fibrotic changes not seen | RUL peripherally. Uptake seen, but only very very subtle CT changes |

| **Study ID** | **000302. Visit 2 scan 06 May 2014** |
| --- | --- |
| CT finding | No change from Visit 1 |
| PET finding | No change from Visit 1 |
| Any areas of fibrotic change on CT where increase radiotracer uptake not seen | No change from Visit 1 |
| Any areas of radiotracer uptake on PET, where CT fibrotic changes not seen | No change from Visit 1 |

| **Study ID** | **000303. Visit 1 scan 13 May 2014** |
| --- | --- |
| CT finding | Severe fibrotic changes in the lower lobes bilaterally, moderate changes in the apical/ant/post segments LUL, severe in the lingular segment LUL, relatively mild changes in the RUL and RML peripherally. |
| PET finding | Very faint activity is present in the areas of fibrotic changes in all lobes. |
| Any areas of fibrotic change on CT where increase radiotracer uptake not seen | No |
| Any areas of radiotracer uptake on PET, where CT fibrotic changes not seen | No |

| **Study ID** | **000303. Visit 2 scan 22 May 2014** |
| --- | --- |
| CT finding | No change from Visit 1 |
| PET finding | No change from Visit 1 |
| Any areas of fibrotic change on CT where increase radiotracer uptake not seen | No change from Visit 1 |
| Any areas of radiotracer uptake on PET, where CT fibrotic changes not seen | No change from Visit 1 |

| **Study ID** | **000304. Visit 1 scan 09 Sep 2014** |
| --- | --- |
| CT finding | Established moderate fibrotic changes LUL>RUL and LLL>RLL. |
| PET finding | Increased activity of varying levels in fibrotic areas of the lung, especially lung bases and upper lobes laterally. |
| Any areas of fibrotic change on CT where increase radiotracer uptake not seen | No, but activity is very low in RUL & LUL fibrotic changes posteriorly. |
| Any areas of radiotracer uptake on PET, where CT fibrotic changes not seen | No |

| **Study ID** | **000304. Visit 2 scan 16 Sep 2014** |
| --- | --- |
| CT finding | No change from Visit 1 |
| PET finding | No change from Visit 1 |
| Any areas of fibrotic change on CT where increase radiotracer uptake not seen | No change from Visit 1 |
| Any areas of radiotracer uptake on PET, where CT fibrotic changes not seen | No change from Visit 1 |

| **Study ID** | **000305. Visit 1 scan 04 Nov 2014** |
| --- | --- |
| CT finding | Moderate changes in all lobes R>L |
| PET finding | Fairly uniform activity in fibrotic areas apart from higher activity in LLL laterally |
| Any areas of fibrotic change on CT where increase radiotracer uptake not seen | No |
| Any areas of radiotracer uptake on PET, where CT fibrotic changes not seen | No, but in LUL peripherally, there is activity in areas of very very subtle CT changes. |

| **Study ID** | **000305. Visit 2 scan 17 Nov 2014** |
| --- | --- |
| CT finding | No change from Visit 1 |
| PET finding | No change from Visit 1 |
| Any areas of fibrotic change on CT where increase radiotracer uptake not seen | No change from Visit 1 |
| Any areas of radiotracer uptake on PET, where CT fibrotic changes not seen | No change from Visit 1 |

| **Study ID** | **000306. Visit 1 scan 19 Nov 2014** |
| --- | --- |
| CT finding | Moderate/severe fibrotic changes all lobes. |
| PET finding | Tracer uptake in all areas of fibrotic changes |
| Any areas of fibrotic change on CT where increase radiotracer uptake not seen | No |
| Any areas of radiotracer uptake on PET, where CT fibrotic changes not seen | No |

| **Study ID** | **000306. Visit 2 scan 01 Dec 2014** |
| --- | --- |
| CT finding | No change from Visit 1 |
| PET finding | No change from Visit 1 |
| Any areas of fibrotic change on CT where increase radiotracer uptake not seen | No change from Visit 1 |
| Any areas of radiotracer uptake on PET, where CT fibrotic changes not seen | No change from Visit 1 |

| **Study ID** | **000307. Visit 1 scan 03 Dec 2014** |
| --- | --- |
| CT finding | Mild peripheral fibrotic changes all lobes apart from lung base where there is moderate changes. |
| PET finding | Very mild activity in the right lung base and upper lobes posteriorly. |
| Any areas of fibrotic change on CT where increase radiotracer uptake not seen | Yes the upper lobes anteriorly.. |
| Any areas of radiotracer uptake on PET, where CT fibrotic changes not seen | No. |

| **Study ID** | **000307 Visit 2 scan 15 Dec 2014** |
| --- | --- |
| CT finding | No change from Visit 1. |
| PET finding | No change from Visit 1. |
| Any areas of fibrotic change on CT where increase radiotracer uptake not seen | No change from Visit 1 |
| Any areas of radiotracer uptake on PET, where CT fibrotic changes not seen | No change from Visit 1 |

| **Study ID** | **000401. Visit 1 scan 25 Jan 2016** |
| --- | --- |
| CT finding | Severe fibrotic changes in all lobes. |
| PET finding | Very mild low level activity throughout both lungs. |
| Any areas of fibrotic change on CT where increase radiotracer uptake not seen | No |
| Any areas of radiotracer uptake on PET, where CT fibrotic changes not seen | No |

| **Study ID** | **000402. Visit 1 scan 16 Mar 2016** |
| --- | --- |
| CT finding | Mild fibrotic changes in the lung bases posteriorly and the very mild changes anteriorly. |
| PET finding | Very low level uptake at the lung bases posteriorly. |
| Any areas of fibrotic change on CT where increase radiotracer uptake not seen | Yes the lung bases anteriorly. |
| Any areas of radiotracer uptake on PET, where CT fibrotic changes not seen | No. |

| **Study ID** | **000402. Visit 2 scan 31 Mar 2016** |
| --- | --- |
| CT finding | No change from Visit 1. |
| PET finding | No change from Visit 1. |
| Any areas of fibrotic change on CT where increase radiotracer uptake not seen | No change from Visit 1. |
| Any areas of radiotracer uptake on PET, where CT fibrotic changes not seen | No change from Visit 1. |

# References
